# Animals in Mortuary Practices of Bronze-Age Pastoral Societies: Caprine Use at the Site of Dunping in Northwestern China

**DOI:** 10.3390/ani13243794

**Published:** 2023-12-08

**Authors:** Yue Li, Ruoxin Cheng, Zexian Huang, Xiaolu Mao, Kexin Liu, Qianwen Wang, Furen Hou, Ruilin Mao, Chengrui Zhang

**Affiliations:** 1 School of Cultural Heritage, Northwest University, Xi’an 710127, China; 2 School of Sociology and Anthropology, Xiamen University, Xiamen 361005, China; 3 Ningxia Provincial Institute of Cultural Relics and Archaeology, Yinchuan 750001, China; 4 Gansu Provincial Institute of Cultural Relics and Archaeology, Lanzhou 730015, China; 5 Department of Anthropology, Harvard University, Cambridge, MA 02138, USA

**Keywords:** China, the first millennium BC, pastoralist, mortuary practice, sheep and goat, age at death, zooarchaeology

## Abstract

**Simple Summary:**

The authors present a preliminary analysis of caprine remains from 70 burials at Dunping, a burial site associated with Bronze-Age pastoralists in northwestern China and featured by the interment of skulls and hooves of domestic ungulates in burial contexts. The minimal impact of weathering on the skeletal elements and the attachment of atlases in skulls suggest that the caprines chosen for burial may have been slaughtered on-site, and that the inhumation of the caprine skulls and hooves occurred shortly after their death. It is likely that the remaining carcasses of the caprines were consumed by or distributed among funeral participants. The mortality profiles indicate the presence of caprines across various age stages at the site, with individuals aged 6–12 months accounting for the largest proportion. This pattern differed from those observed in contemporaneous pastoral societies in the regions nearby.

**Abstract:**

The late second and first millennium BC witnessed extensive economic, cultural, and political exchanges between pastoralists and sedentary farming states in East Asia. Decades of archaeological fieldwork across northern China have revealed a large number of burial sites associated with pastoralists during the first millennium BC. These sites were characterized by the inhumation of specific animal parts in burials, predominantly the skulls and hooves of sheep, goats, cattle, and horses. However, the selection preference for these animals and how they were integrated into the mortuary contexts of these pastoral societies remain poorly investigated. Here, we report a preliminary analysis of caprine remains from 70 burials at the site of Dunping in the southern Gansu region of northwestern China, dated to approximately the seventh to fourth centuries BC. Based on an examination of species composition, post-depositional effects, traces of human alteration, skeletal element representation, and age at death, we discussed the selection, slaughtering, and inhumation of caprines concerning the mortuary practices at the site. Comparisons between Dunping and several other contemporaneous burial sites in neighboring regions, specifically in terms of the mortality profiles, further highlight distinct patterns in the selection of caprines for mortuary purposes among pastoral societies. These differences suggest varying degrees of emphasis placed on the economic and social significance attributed to caprines. Our findings provide new insights into the roles that caprines played in both ritual performances and subsistence practices among pastoralists in East Asia during the first millennium BC.

## 1. Introduction

Animals have been an integral part of human society for millennia, playing a variety of roles in the practical, cultural, emotional, and spiritual aspects of human life [1,2,3,4,5,6]. Examining animal remains from archaeological contexts provides insights into the complex and evolving relationships between humans and animals across different periods [6,7,8,9,10]. Archaeologists have shown significant interest in the use of animals for mortuary purposes. The selection of animals and their inclusion in burials or associated sacrificial contexts often involves a series of specific human actions and rituals [11,12,13]. Investigating how animals were integrated into these contexts provides a window into the interplay between the economic value and ritual significance of animals [6,14,15,16].

The late second and first millennium BC witnessed extensive economic, cultural, and political exchanges between pastoralists and sedentary farming states in East Asia [17,18]. In China, this is evidenced, for example, by the military triumph of the Western Zhou (*ca.* 1045-771 BC) dynasty over raiding pastoralists, as documented in Chinese historical texts and inscriptions on bronze vessels [19]. Frequent incursions of mounted pastoralists from the north and the northwest also triggered military and political disputes with the “Warring States”, particularly the states of Yan, Zhao, and Qin [18,20]. Indeed, the construction of great walls along the borders of these states and their adoption of equestrian skills and mounted archery were in large measure associated with the neighboring pastoralists [21,22,23]. The interactions between pastoralists and agricultural societies had a series of impacts on the geopolitics in northern China prior to the establishment of the first united dynasties by the end of the first millennium BC [20,21,22,24].

Decades of archaeological fieldwork in northern China have revealed a large number of burial sites that are associated with pastoralists on the borders of agricultural states during the first millennium BC. Commonly referred to as the “northern steppe cultures” in Chinese archaeological literature, these burial sites, such as Yuhuangmiao, Yanglang, Wandahu, Majiayuan, and Jinggouzi in present-day Beijing, Ningxia, Gansu, and Inner Mongolia [25,26,27,28,29], exhibit distinct features. They are characterized by the presence of “northern-style” bronze artifacts, including, but not limited to, metal plaques with animal motifs, daggers, and swords [20,25,28,30,31]. Another notable aspect of these sites is the inhumation of specific animal skeletal elements, predominantly the skulls and hooves of sheep, goats, cattle, and horses [28,32]. While the burial of specific animal parts is not uncommon in the archaeological record of ancient China—for example, the interment of pig mandibles in Neolithic burials in both the Yellow River and Yangtze River valleys [33]—the substantial quantity of structured deposits containing skulls and hooves of domestic ungulates is almost exclusively observed in the mortuary practices of the pastoral communities linked to those burial sites.

Previous research has provided insights into the social and political interactions between pastoralists and their agricultural neighbors in the hinterlands of China and other parts of Eurasia during the first millennium BC [20,25]. Animal remains recovered from burials at these sites have contributed to discussions on the mortuary practices, economic patterns, and social structures of pastoral societies in Bronze-Age China [26,29,32,34,35]. However, due to a scarcity of zooarchaeological data, how these animals were selected and integrated into the mortuary contexts remains largely unexplored. We also know little about potential regional variations in the mortuary treatment of animals among different pastoralist communities. Exploring these aspects is crucial to a more holistic understanding of burial customs and human–animal interactions among pastoralists in Bronze-Age China.

In this study, we present an analysis of caprine remains from burials at the first millennium BC site of Dunping in present-day eastern Gansu Province in northwestern China. By analyzing the taxonomic composition of animals within each burial, the representation of skeletal elements, and the age-at-death of caprines, as well as identifying traces of human modification and post-depositional effects, we investigate the use of caprines in mortuary contexts at Dunping and its broad implications for the roles of caprines in subsistence and mortuary practices among pastoral societies during the first millennium BC.

## 2. The Burial Site of Dunping and the *Xi Rong* People

**Figure 1:**
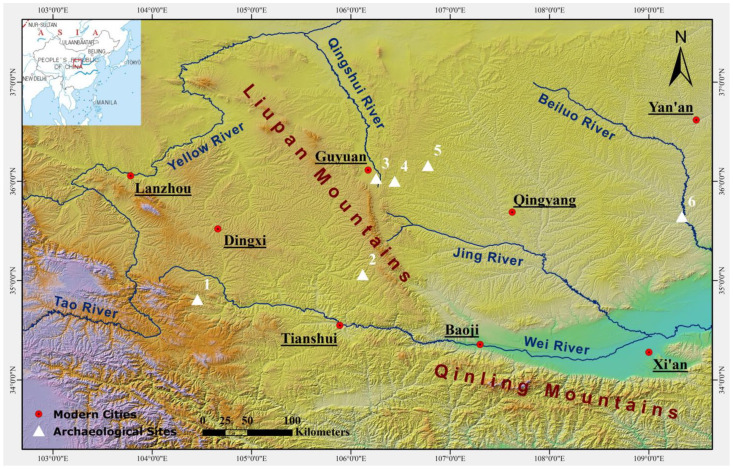
The location of Dunping and Majiayuan, two burial sites associated with *Xi Rong* pastoralists in the first millennium BC in northwestern China. (1) Dunping; (2) Majiayuan; (3) Jiulongshan; (4) Wangdahu; (5) Zhongzhuang; (6) Zhaitouhe. The map was produced in ArcMap 10.7.1. The digital elevation model (DEM) was acquired from the Geospatial Data Cloud site, Computer Network Information Center, Chinese Academy of Sciences (http://www.gscloud.cn, accessed on 18 September 2023).

**Figure 2:**
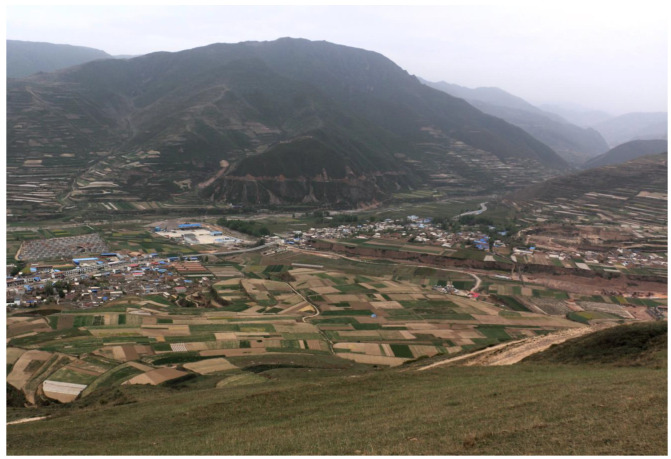
The natural landscape surrounding Dunping (viewing from north to south).

Located on the tableland west of the modern Zhangxian County in Gansu Province (Figure 1), the site of Dunping is flanked by two loess gullies on its eastern and western sides, with the Zhang River, a tributary of the Wei River, flowing through the southern part of the site [36,37] (Figure 2). Due to the damage from looting, the Gansu Provincial Institute of Cultural Relics and Archaeology conducted rescue excavations and surveys beginning in 2014. Fieldwork results suggest that Dunping covers an area of approximately 15 hectares and contains more than 150 burials [38]. Previous archaeological fieldwork in the Zhang River valley has revealed several burial sites linked to the *Xi Rong* people, of which Dunping is to date the largest one of its kind in the region.

**Figure 3:**
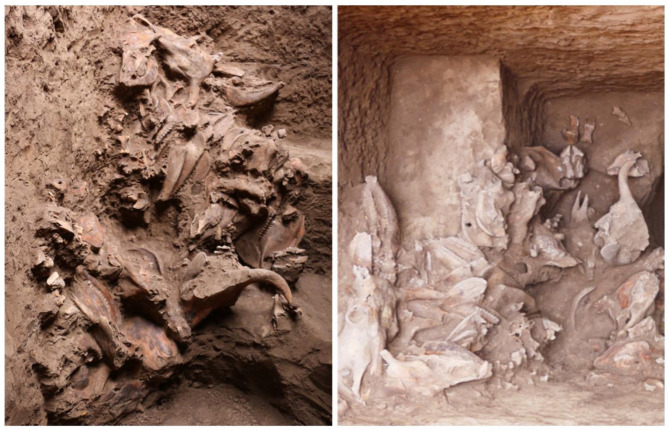
Animal skeletal elements inhumed in burial M26 at Dunping.

All excavated burials at Dunping were pit tombs, each inhumed with a single human skeleton, half of which had side chambers. The deceased were mostly in a supine extended position with their head oriented to the east, of which some were interred in wooden coffins [38,39]. These burials vary in size and the combination of artifacts. The largest burials normally contained one complete chariot and more elaborate artifacts of varying materials. In medium-sized burials, chariot fittings or disassembled chariot elements were interred, which likely symbolized complete chariots. Very few or no artifacts were found in the smallest burials [38,39]. The burials at Dunping also featured the interment of specific skeletal elements of caprines, cattle, and horses. The majority of animal remains were placed in the vertical ramps, with only a small number found in the niches. These animal remains seemed to have no fixed orientations, of which some were positioned in slightly sloped accumulations, while others were scattered within the vertical ramps (Figure 3).

Examining the design of artifacts from excavated burials, such as bronze weapons and chariot fittings, it is evident that, with the exception of a small proportion of burials dated to the later Qin and Han periods, the majority of burials are dated to the mid-Spring and Autumn through the mid-Warring States periods (*ca.* the seventh to fourth centuries BC). Coupled with the location of the site and the inhumation of animal parts, the deceased at Dunping were potentially affiliated with the *Xi Rong* people, as documented in Chinese historical records, such as the *Shiji* (Records of the Grand Historian) [38,39,40,41]. The term *Xi Rong* refers to pastoralists who were active in the Longshan Mountain (also known as the Liupan Mountain) region during the first millennium BC. These individuals engaged in conflicts and exchanges with the Qin State and other agricultural states to their east [27,40,41,42].

Dunping sits approximately 160 km southwest of Majiayuan, another large burial site associated with the pastoralists of the late first millennium BC, which is also considered to belong to the *Xi Rong* people [27,42,43]. While these two burial sites share similarities in terms of the mortuary use of animal parts and their connection with the Qin State, there are notable differences between Dunping and Majiayuan in terms of burial structure and the combination of mortuary objects. Unlike Majiayuan, the burials at Dunping did not feature stair-stepping ramps on the walls and did not contain whole horses. Additionally, sacrificial pits containing partial animals, which were found at Majiayuan, were absent at Dunping [38,39]. These distinctions suggest that these two sites may have been affiliated with different groups of the *Xi Rong* people [40,41]. Furthermore, the social standing of the deceased at Majiayuan appears to have been higher than that of the Dunping pastoralists, potentially indicating varying levels of status and wealth between the two groups.

## 3. Materials and Methods

In this study, we analyzed caprine remains recovered from 70 burials at Dunping, dating from the mid-Spring and Autumn through the late Warring States periods (*ca.* the seventh to fourth centuries BC). Most of the burials had been disturbed by looting activities to varying degrees, resulting in a proportion of animal skeletal elements being discovered in the looting holes of 20 burials. Bearing this in mind, the animal remains recovered from these burials during excavation might not entirely represent the complete assemblage initially interred in each burial, although previous excavations of burials of the Xiongnu period (*ca.* second century BC to second century AD) in Mongolia show that the inhumed animal remains often remain intact even if the burials were looted, resulting in the disturbance or loss of human skeletons and burial objects [14]. While it is believed that the animal remains from looting holes were originally part of the assemblages with each burial at Dunping, we exclude these specimens from further analysis and only briefly present them in the Results section.

Caprine remains from the 70 examined burials exhibited morphological characteristics consistent with domestic sheep (*Ovis aries*) or goats (*Capra hircus*). By the time of Dunping, domestic sheep and goats had already been integrated into the animal economies of the middle and upper reaches of the Yellow River valley for at least a millennium. These herbivores have been identified in the animal assemblages from residential and ritual contexts at numerous late Neolithic and Bronze-Age sites associated with both farming societies and pastoralists across northern China [44,45,46,47]. Therefore, we believe that the caprines found at Dunping were domestic sheep and goats. The differentiation between sheep and goats was based on previous morphological studies [48,49,50] and both archaeological and modern reference collections stored in the Zooarchaeology Lab of Northwest University in Xi’an, China. We primarily used the frontal suture, pyramidal eminence region, and mandible with dentition to separate sheep from goats. Apart from complete caprine skulls that were readily separable, we grouped fragmented or isolated craniums and mandibles as the category of “sheep/goat (*Ovis aries*/*Capra hircus*)” if distinguishable morphological features were absent.

Besides morphological features, we recorded identifiable traces of human alteration [3,5] and counted the number of atlases and hyoids in these burials. We also documented post-depositional effects on the skeletal elements to evaluate their preservation [51].

We calculated the number of identified specimens (NISP) and the minimum number of individuals (MNI) of caprines from the burials [3,5]. In particular, the MNI was calculated by adding up the minimum number of identified caprine individuals for each burial.

We estimated the age at death of these caprines and reconstructed their mortality profiles primarily based on the eruption and wear of the mandibular teeth [52,53,54]. However, it is important to note that not all caprine individuals eligible for age estimation could be definitely assigned to a single age stage, such as “6–12 months”, due to variations in tooth preservation. In several instances, the absence or breakage of specific teeth necessitated the use of multiple stages or lower/upper age limits, such as “36–48 months/48–72 months” and “above 18 months”. Consequently, these individuals, despite having age estimations, were not included in the analysis.

Statistical test was performed in IBM SPSS Statistics, version 29.0.0.0 (241).

## 4. Results

### 4.1. Species Representation and Skeletal Elements

The identified caprine remains from the 70 examined burials added up to 4427 pieces by NISP (Table 1), of which 1532 were identified as sheep (*Ovis aries*), 1388 as goats (*Capra hircus*), and 1507 as sheep/goats (*Ovis aries*/*Capra hircus*). Based on the highest count of each type of skeletal element from each burial, these caprine remains represented at least 1511 individuals (Table 1). Specifically, 576 (38.1%) of these individuals were sheep, 544 (36.0%) were goats, and 391 (25.9%) were sheep/goats.

**Figure 4:**
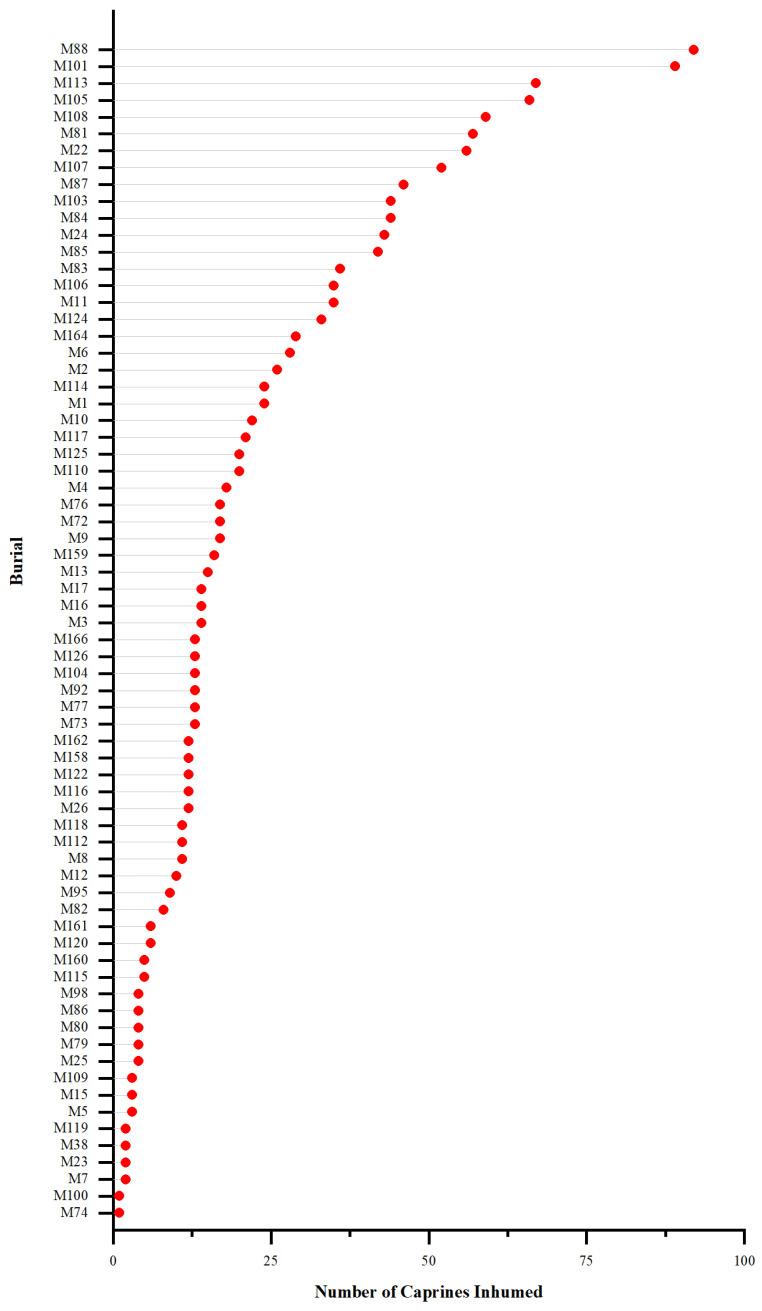
The number of caprines (by MNI) interred in the 70 burials at Dunping, dated to the mid-Spring and Autumn to the late Warring States periods (*ca.* the seventh to third centuries BC).

For each burial, the number of caprines (by MNI) interred ranges between 1 and 92, averaging 21.6 (Figure 4). The percentage of caprines in the animal assemblage from each burial ranges between 8.3% and 100.0% (averaging 68.6%). A total of 58 burials contained caprines that took up no less than 50.0% of all animals inhumed. For example, 130 herbivore individuals were found in burial M88, of which 70.8% (*n* = 92) were caprines. Among the 70 burials examined, 35 (50.0%) contained caprines, cattle, and horses, 23 (32.9%) had caprines and cattle, and two (2.9%) had caprines and horses. Ten (14.3%) burials contained exclusively caprines. Cattle and horses, especially the latter, were less frequently observed in burials at Dunping.

**Figure 5:**
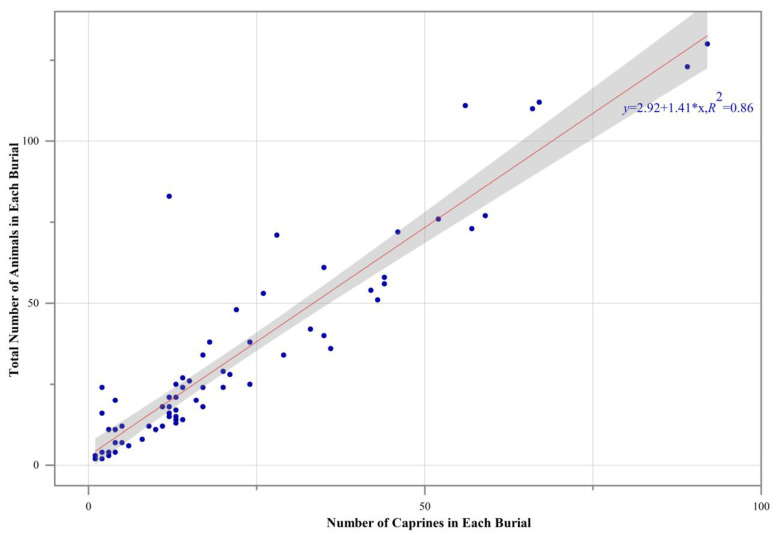
The correlation between the number of caprines and the total number of animal individuals in each burial at Dunping, based on unary linear regression. Each blue dot stands for one specific burial. The gray band represents 95% confidence interval.

A unary linear regression indicates a relatively strong correlation between the number of caprines and the total number of animal individuals in each respective burial (Figure 5), suggesting that caprines might represent the whole animal assemblage within each burial.

In terms of the representation of skeletal elements, the caprine remains inhumed in these burials predominantly consisted of skulls and phalanges, alongside hundreds of atlases. Only a minimal proportion (less than 0.1% of the total assemblage) of scapulae, metapodials, and carpals were recovered from these burials. In total, 432 atlases (by MNI) were found in 60 of 70 examined burials. This does not count in atlases from looting holes and isolated ones that could not be attributed to specific caprine individuals. Phalanges were commonly found adjacent to or beneath the skulls, not in their anatomical arrangements.

It is worth mentioning that a total of 222 caprine remains (by NISP) were found in the looting holes of 19 examined burials. Skulls took up the largest proportion of these remains (*n* = 169, 76.1%), followed by atlases (*n* = 23, 10.4%) and phalanges (*n* = 19, 8.6%). The remaining remains were forelimbs (scapulae, humeri, radii, and metacarpals), hindlimbs (tibiae and metatarsals), and vertebrae. While the looting holes contained a relatively higher proportion of limb bones, the composition of skeletal elements found within these holes mirrors that of the remains recovered from the burials.

### 4.2. Potential Layered Deposits of Animal Remains in Burials

In 27 out of the 70 examined burials, the animal remains in the vertical ramps exhibited a layered arrangement, with the number of animals in each layer varying from two to twelve (by MNI). To illustrate potential layered animal interments at Dunping, three specific burials were selected for demonstration.

Burial M22 had 12 layers of animal remains, of which caprines took up over 50.0% of the skeletal elements across nine layers. All caprines remains in this burial consisted solely of skulls, phalanges, and atlases (as outlined in Table 2).

Burial M88 contained the largest quantity of animal remains among the 70 examined burials. These animal remains seemed to be inhumed in six layers, with caprines taking up over 50.0% of the skeletal elements in five of these layers. Like M22, all caprine remains found in M88 were skulls, phalanges, and atlases (Table 3).

Burial M125 housed an intermediate number of inhumed animals, arranged in four layers. In each of these layers, caprines accounted for over 50% of the skeletal elements. With only one exception, all caprine specimens in this burial were skulls, phalanges, and atlases (Table 4).

### 4.3. Traces of Human Modification

**Figure 6:**
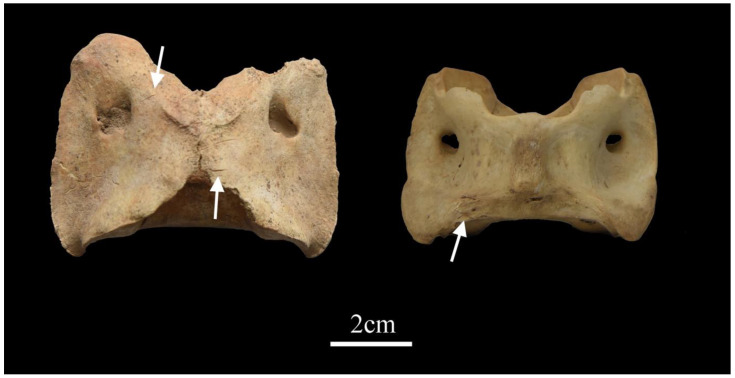
Cut marks on caprine atlases unearthed from burials M107 (**left**) and M88 (**right**).

We observed discernable traces of human alteration on six atlases, including two sheep, one goat, and three sheep/goats. These marks are manifested as cut or incision marks, primarily located on the articular surface and dorsal surface of the atlases (Figure 6). Besides atlases, we only identified human-induced marks on a few mandibles (*n* = 5) and phalanges (*n* = 5); all were consistent with cuts or incisions.

### 4.4. Post-Depositional Effects

**Figure 7:**
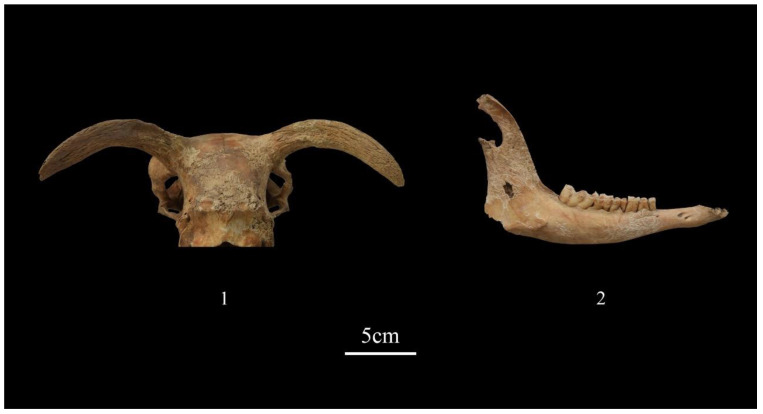
Moderate weathering on caprine remains from burials at Dunping. (1) A sheep skull from burial M83; (2) a goat right mandible from burial M85.

The animal remains recovered from the burials at Dunping were well preserved. Except for deformations and breakages caused by post-depositional effects such as bioturbation, burial collapse, or digging, no traces of rodent gnawing, carnivore chewing, or burning were identified on the analyzed caprine remains. Out of all caprine remains, 315 skeletal elements, constituting 7.1% of the total, exhibited varying degrees of weathering (Figure 7). Among them, 27 displayed very light weathering with mosaic cracking (similar to Stage 1 in Behrensmeyer’s system), while 102 exhibited more severe weathering with a rough or fibrous surface (similar to Stages 3–4 in Behrensmeyer’s system). The remaining 186 skeletal elements displayed degrees of weathering in between, with slight flaking (similar to Stage 2 in Behrensmeyer’s system).

### 4.5. Mortality Profiles

Due to a pre- and post-mortem loss of teeth, breakage, and lack of diagnostic features, we were not able to estimate the age at death of 55 caprine individuals. Age-at-death estimates were determined for 1456 caprine individuals with preserved lower dentitions, including 567 sheep, 538 goats, and 351 sheep/goats. These individuals accounted for 96.4% of all caprines recovered from the 70 examined burials. A total of 1367 caprines were successfully assigned a single age stage.

**Figure 8:**
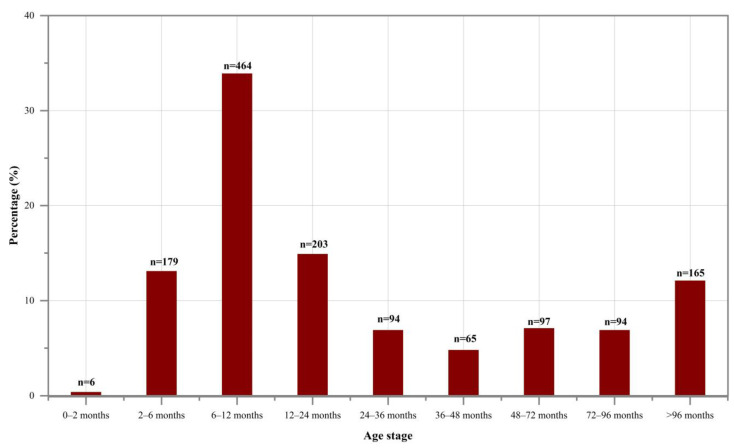
Age structure for 1367 caprine individuals (viewing all caprines as a collective group).

If we consider these 1367 caprines as a collective group regardless of species, roughly one-third of the individuals were aged below 6–12 months (Figure 8). The subsequent age stages, listed in descending order of representation, were “12–24 months”, “2–6 months”, and “>96 months”. The percentage of caprines falling into each age stage between 24 and 96 months was notably low, and individuals that died within 0–2 months of age were exceedingly rare across the examined burials.

**Figure 9:**
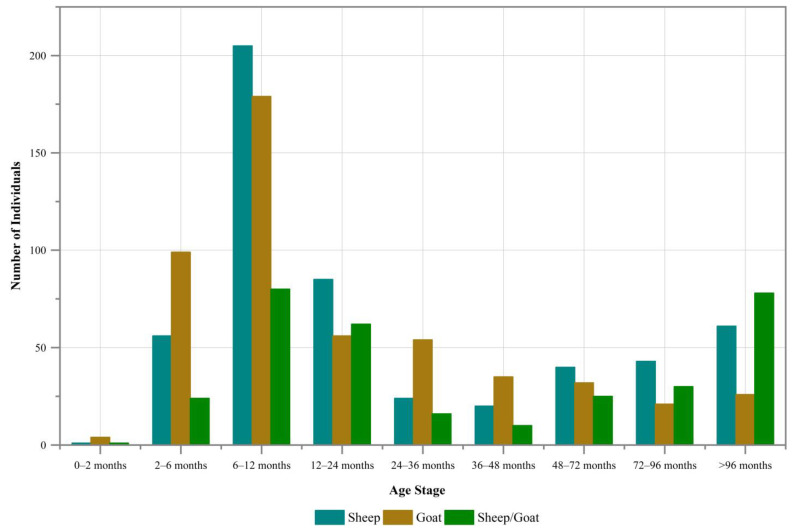
Age structure for 1367 caprine individuals (analyzing sheep, goat, and sheep/goat separately).

The mortality profiles of these caprines, when analyzed separately by species, reveal a similar pattern, where the largest proportion in each respective group consists of individuals aged 6–12 months (Figure 9). There were some minor differences observed for specific age stages among the groups. Notably, the sheep/goat group exhibited a relatively higher proportion of elder caprines (“>96 months”) and a lower proportion of young individuals (“6–12 months”), indicating that this group had more mature individuals and fewer young ones compared to the other two groups. However, in general, the age-at-death structures remained similar across the three species groups studied. The results of the chi-square test also indicate that there is no statistically significant difference in the age-at-death structures among the three species groups.

## 5. Discussion

While the current absence of residential features in the archaeological record limits our understanding of specific aspects of everyday life in the Dunping community, the widespread interment of numerous sheep and goats, alongside cattle and horses, remains indicative of a reliance on pastoralism [55]. Indeed, the adaptability of domestic caprines to various climates and terrains, coupled with their relatively low maintenance requirements, made them well suited for Bronze-Age pastoralists on the western edge of the Loess Plateau, a region characterized by a semi-arid climate and lower annual precipitation. Miller suggests that the presence of animal remains in burials of the Xiongnu period, also in the form of partial bodies, not only reflects the role of herd animals in subsistence economies but also carries ritual and social significance among the Xiongnu communities [14]. This perspective similarly applies to the Dunping case, where the inclusion of specific skeletal elements in burials emphasizes their significance beyond their mere involvement in subsistence practices.

### 5.1. The Slaughtering and Inhumation of Caprines as Part of the Mortuary Practices at Dunping

The caprines inhumed in burials at Dunping, alongside cattle and horses, may have been personal possessions of the deceased during their lifetime, serving as essential sources of meat and secondary products [14]. It is also likely that the animals were offered as condolence gifts by mourners, who could have included family members, other households of the community, or individuals from other communities, signifying social connections between funeral participants and the deceased.

Skulls and phalanges, containing relatively less meat than other parts (e.g., ribs), may have served as symbolic representations of whole animals for the deceased [14]. Alternatively, if complete caprines, irrespective of their sources, were slaughtered on-site during funeral ceremonies, the remaining carcasses were likely either consumed by funeral participants in the form of communal consumption (e.g., feasts) or distributed among attendees as an expression of appreciation [12,56,57,58]. Indeed, many of the caprine individuals interred in the examined burials were of an age optimal for yielding quality meat. In either scenario, the consumption or sharing of these animals may have contributed to reinforcing social bonds within specific groups and the wider community [14,59,60]. As previously mentioned, skeletal elements in several burials at Dunping exhibited a layered arrangement. These observations, unless attributed to looting activities, suggest that the interment of animal remains may have occurred gradually, possibly corresponding to different steps of specific funerary rituals. Alternatively, each layer of animal remains could represent offerings from different groups of funeral participants.

At Dunping, the animal remains may have been interred as offerings, possibly intended as sustenance for the deceased in their afterlife. These skeletal elements could also have symbolized personal wealth, reflecting either the possessions of the deceased during their lifetime or indicating the quality of the deceased’s interpersonal connections. The analysis of human remains and the organization of excavated burial data are still ongoing. Based on the limited information available, a potential correlation appears to exist between the number of interred animal individuals and the burial’s status (e.g., size and included objects, as previously mentioned). For example, burial M22, which contained wooden chariot wheels, bronze chariot fittings, golden ornaments, and both inner and outer coffins, had more than one hundred animal individuals, approximately half of which were caprines. In comparison, burials such as M1 and M3, smaller in size than M22 and without chariot wheels [38], contained an apparently smaller number of animals. That being said, this tentative inference remains to be confirmed in future research when all burial data are available.

The pattern of weathering and the presence of certain skeletal elements provide clues on how caprines were slaughtered and inhumed in burials at Dunping. The atlanto-occipital joint, formed by the anterior articular concave of the atlas and the occipital condyle of the skull, is covered by ligaments and fibrous tissue membranes. These structures allow for head flexion and extension while protecting the spinal cord [61]. Depending on the method of slaughter, the atlas can either remain attached to the skull or be severed from it. The time it takes for the atlas to separate from the skull after slaughtering may vary due to factors such as processing methods and environmental conditions. Nevertheless, the separation typically occurs relatively quickly due to the loss of blood circulation and the breakdown of connective tissues. This process can be accelerated in warmer environments or if the head is subjected to mechanical forces during transportation or processing.

At Dunping, nearly one-third of caprine individuals examined had their atlases attached to the skulls in their original anatomical positions upon excavation, and the phenomenon was observed in 60 out of the 70 examined burials and was also noted in horses and cattle. Such occurrences are not uncommon in the archaeological record of northwestern China and beyond. For example, atlases are found attached to animal skulls in burials at the aforementioned site of Wangdahu [29]. In Bronze-Age horse burials and burials of the Xiongnu period in Mongolia, the skulls of caprines and horses are also found with atlases and/or axes [14,62]. At Dunping, at least a proportion of caprines were killed without severing the atlas, and these individuals were supposed to be buried shortly after culling. This inference is further supported by the presence of hyoid bones. The hyoid bone, suspended by ligaments and not directly articulating with other bones, would normally become detached from surrounding tissues during the slaughtering process unless intentionally preserved. The presence of hyoid bones in the caprine assemblages from Dunping, albeit in small numbers (*n* = 119), indicates that the corresponding individuals were interred in burials relatively soon after death. The limited degree of weathering also implies that many of the examined caprine remains did not experience prolonged exposure to the air. That being said, the skulls and hooves that showed a high degree of weathering could also be preserved and displayed for a period before being chosen for burial. Indeed, in ethnographic records of Southeast Asia, animal parts, such as the horns of water buffaloes and the mandibles of pigs from communal events, are kept and displayed in specific locations to show the ability of sponsorship or prestige [56,63].

It is noteworthy that there is not a high frequency of cut marks observed on the atlases and other skeletal elements. This could indicate that the individuals responsible for caprine culling were highly skilled and well versed in the anatomical features of caprines, thereby leaving fewer traces of slaughtering activities. It is also possible that the caprines were processed using methods that did not leave prominent cut marks.

### 5.2. Mortuary Use of Caprines among Pastoralists of the First Millennium BC in Northwestern China

Previous research suggests that a high mortality rate of caprines aged 1–3 years indicates their utilization for meat consumption, while a focused slaughter at 6–12 months could signify a preference for tender meat [52,64]. At Dunping, the apparent prevalence of juvenile caprines in burials corresponds with the age profile indicative of caprine consumption as a primary protein source at settlement sites across Neolithic and Bronze-Age Eurasia [64,65,66]. For example, at settlement sites in neighboring regions contemporaneous with Dunping, such as Yucun [67] and Majiaoping [68], affiliated with farming societies (specifically the Qin people), individuals aged one year or younger, particularly those between 6 and 12 months, constituted the largest proportion. Caprines older than 8 years were also present at Yucun and Maojiaoping.

To gain better insight into how caprines were used in mortuary practices among pastoralists in northwestern China during the first millennium BC, we compared our findings with data from several contemporaneous sites in neighboring regions, with a specific emphasis on Wangdahu [29], Zhongzhuang [29], Jiulongshan [29], and Zhaitouhe [69]. The first three sites are located in present-day southern Ningxia, approximately 200 km to the northeast of Dunping, while Zhaitouhe is located in northern Shaanxi, approximately 450 km to the northeast of Dunping. As partly mentioned before, these sites were associated with pastoralists in the first millennium BC and were featured by the use of animal skulls and hooves in mortuary contexts, despite slight differences from Dunping in terms of the burial structure and the location of inhumed animal remains. Among burial sites of a comparable nature, Wangdahu, Zhongzhuang, Jiulongshan, and Zhaitouhe were the few with the age data of animal remains available in the published archaeological literature. In this study, we reanalyzed the published zooarchaeological data from these sites and compared them with the data obtained from Dunping.

**Figure 10:**
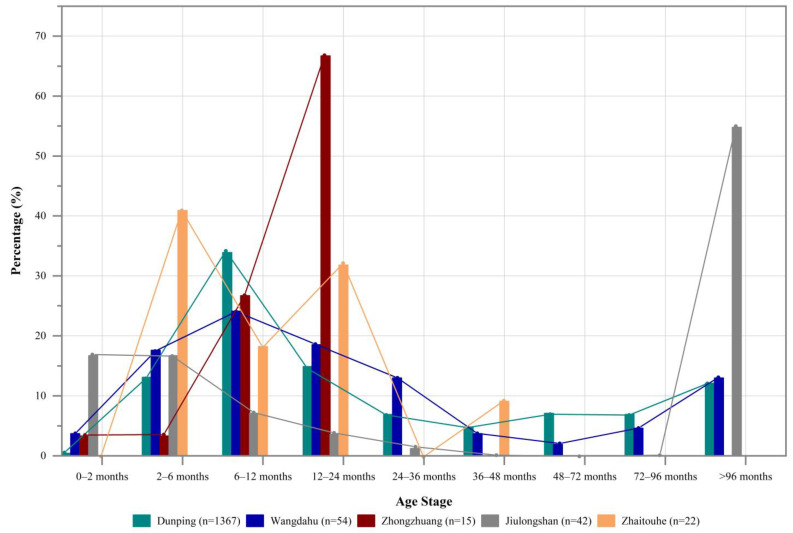
Age structure for caprines from Dunping, Wangdahu, Jiulongshan, Zhongzhuang, and Zhaitouhe (viewing all caprine individuals as a group at each site).

At Wangdahu, seven out of eight excavated burials contained animal skulls and hooves, which were placed in separate layers. These skeletal elements represented at least 148 individuals, including 95 caprines, 21 cattle, and 32 horses. Age-at-death estimates were available for 91 caprines. Approximately 36.3% (*n* = 33) of these individuals were below 12 months of age. While the age of most other caprines could only be estimated as older than 12 months, at least 16 (17.6%) were aged 1–3 years, and six (6.6%) were aged above 8 years [29]. Similar to Dunping, caprines of all age stages were included in burials at Wangdahu, with the assemblage primarily consisting of individuals aged between 2 months and 3 years (Figure 10). The mortality profiles for caprines from both Dunping and Wangdahu appear to reflect a pattern of meat acquisition.

Zhongzhuang presents a slightly different scenario. Animal remains were found in a single burial at the site, representing a total of 56 individuals, including 44 caprines, seven cattle, and four horses. Age-at-death estimates were available for 16 out of the 44 caprine individuals. Among these, four (57.5%) were below one year old, and nine (22.5%) were aged 1–2 years. Only four caprines could be assigned an age above one year, and no aged individuals were recovered from this particular burial [29]. The mortality profile for caprines from Zhongzhuang indicates a subsistence pattern, although the assemblage appears to contain more caprines aged 1–2 years and fewer aged 6–12 months compared to Dunping and Wangdahu (Figure 10).

Jiulongshan, on the other hand, showed another pattern. In total, 180 animal individuals were found in 11 burials, consisting of 169 caprines, 17 cattle, and three horses. Regarding the age at death of the 119 caprine individuals, 43 were younger than 12 months, two were between 1 and 3 years old, 23 were above 8 years, and the remaining 51 could only be identified as older than 12 months. Among the individuals younger than 12 months, at least 14 were younger than 6 months [29]. If we exclude the 51 individuals with a wider age range, over half of the caprines were younger than 12 months, while approximately one-third (33.8%) were aged individuals (over 8 years old). Unlike Dunping, Wangdahu, and Zhongzhuang, the mortality profile for caprines from Jiulongshan exhibited a higher proportion of aged and very young individuals (Figure 10).

In the case of Zhaitouhe, at least 112 animal individuals were recovered from 45 excavated burials, of which 28 (62.2%) had caprine remains. A total of 22 caprine individuals had age estimations based on the eruption and wear of lower teeth. Nearly half (40.9%) of these caprines were 2–6 months of age, followed by individuals aged 12–24 months, 6–12 months, and 36–48 months, which took up 31.8%, 18.2%, and 9.1%, respectively (Figure 10). The predominance of individuals younger than 2 years old corresponds with the optimal acquisition of meat. The absence of caprine individuals above 4 years of age further implies that the mortality profile of caprines chosen for mortuary purposes was similar to that of individuals used for subsistence purposes, which was different from Dunping and the three sites located in southern Ningxia.

A Friedman’s two-way ANOVA test highlights statistically significant differences in the mortality profiles of caprines from the five investigated sites (*x*^2^ = 25.234, *df* = 4, *p* < 0.001). Further pairwise comparisons indicate that Dunping significantly differs from Zhongzhuang, Jiulongshan, and Zhaitouhe (*p* < 0.001), while showing no significant difference from Wangdahu (*p* = 0.101). The differences between Zhongzhuang, Jiulongshan, and Zhaitouhe are statistically significant (*p* = 0.551, *p* = 0.823, and *p* = 0.709, respectively). While Wangdahu shows statistical similarity to Jiulongshan (*p* = 0.086), it differs from Zhaitouhe (*p* = 0.037) and Zhongzhuang (*p* = 0.021).

Overall, the age structures observed in Dunping and Wangdahu seem to align with the composition of animal herds used in subsistence practices. However, Zhongzhuang and Jiulongshan, in particular the latter, exhibited an emphasis on specific age stages, notably lacking sub-adults and elder caprines, while Zhaitouhe showed a clear preference for young caprines. These biases toward particular mortality profiles within a specific species have frequently been interpreted as indicative of the ritual manipulation of animals [3,70]. Although our findings might be influenced by variations in sample sizes across the five investigated burial sites, the analysis of caprine mortality profiles reveals distinct patterns in the mortuary treatment of caprines among pastoral communities in northwestern China during the first millennium BC. Future research will focus on understanding the extent to which these variations were influenced by diverse factors such as subsistence strategies, cultural practices, community structure, and environmental conditions across these pastoral societies.

## 6. Conclusions

The caprine remains recovered from Dunping represent, to date, the most extensive collection of its kind from burial sites associated with Bronze-Age pastoralists in northwestern China. The mortality profiles of these caprines, their relatively rapid interment, and the presence of atlases provide compelling evidence of deliberate selection, culling, and burial. These practices underscore the significant roles that caprines fulfilled in both the subsistence and mortuary aspects in the Dunping community. The comparative analysis between Dunping and contemporaneous burial sites provides insights into the variations in the mortuary use of caprines among pastoral societies in northwestern China during the first millennium BC.

Coupled with the examination of artifact assemblages and human remains from the burials, future research that integrats stable isotope analysis and genetic analysis has the potential to unveil the feeding and management strategies for the animals, as well as their origins, mobility, and genetic relationships, thereby providing further insights into how domestic ungulates were incorporated into the mortuary practices of Bronze-Age pastoralists in East Asia.

## Figures and Tables

**Table 1:** The proportions of sheep and goats in the caprine assemblage from the 70 burials. Abbreviations: NISP = number of identified specimens; MNI = minimum number of individuals.

Species	NISP	MNI	% of Total MNI of Caprines from the 70 Burials	% of Total MNI of Animals from the 70 Burials
Sheep	1532	576	38.1	24.7
Goat	1388	544	36.0	23.3
Sheep/Goat	1507	391	25.9	16.8
Total	4427	1511	100.0	64.7

**Table 2:** The composition of animals and skeletal elements in each layer of infillings of burial M22.

Layer	Animal Species (by MNI)			Caprine Skeletal Element (by NISP)					
	Horse	Cattle	Caprine	Cranium	Mandible	Isolated Tooth	Atlas	Phalanges	Others
Layer 1	1	4	4	4	7	1	4	13	0
Layer 2	1	4	0	0	0	0	0	0	0
Layer 3	0	11	7	7	12	0	1	1	0
Layer 4	1	0	0	0	0	0	0	0	0
Layer 5	0	0	3	3	6	0	2	7	0
Layer 6	0	2	7	6	13	0	3	5	0
Layer 7	0	4	10	8	19	0	2	2	0
Layer 8	0	9	6	4	12	0	3	11	0
Layer 9	1	7	7	7	7	0	1	6	0
Layer 10	6	3	0	0	0	0	0	0	0
Layer 11	0	0	4	2	6	0	2	0	0
Layer 12	0	1	0	0	0	0	0	0	0
Total	10	45	48	41	82	1	18	45	0

**Table 3:** The composition of animals and skeletal elements in each layer of infillings of burial M88.

Layer	Animal Species (by MNI)			Caprine Skeletal Element (by NISP)					
	Horse	Cattle	Caprine	Cranium	Mandible	Isolated Tooth	Atlas	Phalange	Others
Layer 1	0	5	15	14	25	0	1	6	0
Layer 2	0	1	1	1	2	0	0	1	0
Layer 3	0	3	2	1	3	0	0	0	0
Layer 4	0	9	29	29	49	0	15	9	0
Layer 5	4	14	27	24	47	0	12	66	0
Layer 6	0	2	18	17	32	0	8	13	0
Total	4	34	92	86	158	0	36	95	0

**Table 4:** The composition of animals and skeletal elements in each layer of infillings of burial M125.

Layer	Animal Species (by MNI)			Caprine Skeletal Element (by NISP)					
	Horse	Cattle	Caprine	Cranium	Mandible	Isolated Tooth	Atlas	Phalange	Others
Layer 1	1	2	7	5	12	1	4	6	1
Layer 2	0	0	6	4	11	1	0	5	0
Layer 3	0	3	3	2	6	6	3	0	0
Layer 4	2	1	4	2	7	0	0	0	0
Total	3	6	20	13	36	8	7	11	1

## Data Availability

All relevant data are included in this manuscript.
